# Expanding NIST Calibration of Fluorescent Microspheres for Flow Cytometry to More Fluorescence Channels and Smaller Particles

**DOI:** 10.3390/ma13184111

**Published:** 2020-09-16

**Authors:** Paul DeRose, Linhua Tian, Elzafir Elsheikh, Aaron Urbas, Yu-Zhong Zhang, Lili Wang

**Affiliations:** 1 Biosystems and Biomaterials Division, National Institute of Standards and Technology (NIST), Gaithersburg, MD 20899, USA; linhua.tian@nist.gov (L.T.); elzafir.elsheikh@nist.gov (E.E.); lili.wang@nist.gov (L.W.); 2 Chemical Sciences Division, National Institute of Standards and Technology (NIST), Gaithersburg, MD 20899, USA; aaron.urbas@nist.gov; 3 Protein and Cell Analysis, Thermo Fisher Scientific, Eugene, OR 97402, USA; Yu-Zhong.Zhang@thermofisher.com

**Keywords:** beads, calibration, cytometer, flow cytometry, fluorescence channels, fluorophores, microspheres, nanospheres, NIST, reference materials, standards

## Abstract

The National Institute of Standards and Technology (NIST), the National Institutes of Health (NIH) and other industry stakeholders have been working together to enable fluorescence intensities of flow cytometer calibration beads to be assigned quantitative equivalent reference fluorophore (ERF) values with high accuracy and precision. The ultimate goal of this effort is to accurately quantify the number of antibodies bound to individual living cells. The expansion of this effort to assign ERF values to more than 50 fluorescence channels and particles with diameters ranging from 10 μm down to 80 nm is reported here.

## 1. Introduction

A Flow Cytometry Quantitation Consortium has been formed between calibration microsphere/nanosphere (bead) manufacturers, NIH, NIST and other industry stakeholders [1] to enable fluorescence intensity value assignments of cytometer calibration beads in units of equivalent reference fluorophores (ERF) [2]. Flow cytometry (FCM) is used routinely in clinical diagnostics to differentiate and count cells in blood and other body fluids by tagging antigens on cell surfaces, or within cells with fluorescently labeled monoclonal antibodies. The fluorescence intensity of these antibodies is employed as a measure of the antigen expression level, indicative of the presence of a functional gene. The ultimate goal of ERF value assignments is the quantitation of the number of antibodies bound per cell (ABC) for a particular target antigen of interest, such as CD4 or CD19. The ERF scale is an instrument-independent fluorescence intensity scale which is directly proportional to an ABC scale, but the absolute relationship between the two scales is dependent on the properties of both the fluorescent label and the antibodies [3,4]. Before the ABC values of samples can be determined, the fluorescence channels being used in an assay must be calibrated, typically using calibration beads with known fluorescence intensities. Cytometer calibration is a prerequisite for ensuring instrument performance, proper compensation, and assay standardization [5]. Fluorophore solutions of known concentration, such as Standard Reference Material (SRM) 1934 [6], are used to assign fluorescence intensities to the beads in units of ERF. This is done by measuring the fluorescence intensity of both the reference fluorophore solution and the bead suspension using a fluorescence spectrometer, not a flow cytometer, under the same instrument conditions.

Standard Reference Material 1934, comprised of four reference fluorophores, is used at NIST to assign ERF values to calibration beads for 29 fluorescence channels with a spectral emission range from 425 to 810 nm using three excitation lasers (405, 488 and 633 nm). At least 15 of these channels are independent of each other, meaning that there is no significant overlap between emission ranges for channels with the same excitation wavelength. Here, we report the addition of three more laser wavelengths at 375, 561 and 808 nm, and three more reference fluorophores. These additions have enabled NIST to increase the number of assigned fluorescence channels to more than 50, with at least 23 of them being independent of each other.

In order to assign the mean fluorescence intensity for a single calibration bead, the bead concentration of the calibration bead suspension needs to be measured in addition to its fluorescence intensity. NIST uses light obscuration (LO) as the primary measurement technique for number concentration determinations of beads with mean diameters ranging from 2 to 10 μm. This size range corresponds to that of cells typically measured with FCM. Flow cytometry with an internal counting standard has also been used as a secondary technique to confirm the LO values for number concentration. Calibration beads need to be approximately the same size as the biological particles to be characterized in order to accurately calibrate a flow cytometer for the sample of interest. LO gives inaccurate concentrations for particles with diameters less than 2 μm, which excludes its use for the characterization of submicrometer calibration beads.

Extracellular vesicles (EVs) are submicrometer biological structures that have recently inspired heightened research interest for characterization, using a variety of techniques. They can be released by cells and are suspected to mediate intercellular communication. Even though they are smaller than cells, they exhibit many of the same antigens as their parental cells. FCM can potentially be used to distinguish between EV subtypes and evaluate their respective biological activities using antibody-binding strategies in the same way that is presently being done for cells. The characterization of these nanobioparticles with FCM will require similar particles/bioparticles as instrument performance and counting controls. This presents new challenges for particle characterization and ERF value assignment under the consortium. Here, we report the preliminary results for measuring particle concentrations of submicrometer beads down to 80 nm using various techniques.

## 2. Materials and Methods

### 2.1. New Reference Fluorophores

All three of the new reference fluorophores were produced by Molecular Probes^®^ (Life Technologies/Thermo Fisher Scientific, Eugene, OR, USA). Pacific Orange (PO) was produced as Pacific Orange triethylamine salt SKU # MT38404, Lot # EN0061-023-YH, and determined by the manufacturer to be about 99% pure using high performance liquid chromatography (HPLC) with absorbance detection at 398 nm. Alexa Fluor 700 (AF700) was produced as Alexa Fluor 700 carboxylic acid, tris (triethylammonium salt) SKU # MT35335, Lot # 1967899, and determined by the manufacturer to be about 99% pure using HPLC with absorbance detection at 700 nm. Alexa Fluor 750 (AF750) was produced as Alexa Fluor 750 carboxylic acid, tris (triethylammonium salt) SKU # MT35338, Lot # 1877862A, and determined by the manufacturer to be about 99% pure using HPLC with absorbance detection at 750 nm. The HPLC purities were expressed in mole percent.

The purity of all three dyes was determined by NIST using quantitative ^1^H-NMR (qNMR). HPLC was used as supporting evidence to help assign the identities of impurities. Each dye was dried in an oven before being weighed and dissolved in dimethyl sulfoxide (DMSO). The resulting fluorescent reference solutions for internal NIST use were assigned reference values for concentration in units of mass of dye per mass of solution, using gravimetry and absolute dye purity. The dye solutions were put into flame-sealed ampoules under argon gas in 2 mL aliquots. The concentrations of dye solution determined from measured masses of dye and solvent were adjusted for purity. The corresponding uncertainties include those related with both the purity and mass measurements.

#### 2.1.1. qNMR

Quantitative 1H-NMR with an internal standard was used to determine the absolute purity for the solid samples of PO, AF700 and AF750. The internal standard used for all samples was 1,3,5-trimethoxybenzene (Sigma-Aldrich, TraceCERT^®^, Lot BCBW3670). The certified mass fraction of this standard is traceable to the International System of Units (SI) through NIST PS1 Primary Standard for qNMR (Benzoic Acid) [7,8,9].

#### 2.1.2. HPLC

The fluorescent dyes analyzed here are expected to have impurities that are either aromatic compounds, such as starting materials and intermediates that did not fully react to become the final dye product, or solvent residues that were not removed completely during the organic syntheses that produced the dyes. The separation of mixtures of organic compounds with similar structures can often be done most effectively using reversed-phase HPLC. The three fluorescent dyes and their aromatic impurities were separated and detected using HPLC with UV absorbance detection. All impurities were detected with the greatest sensitivity at an absorbance wavelength of 262 nm. Therefore, absorbance at 262 nm was selected to determine purity with HPLC. Chromatograms of each solution and the solvent were collected. The solvent chromatogram was subtracted from the solution chromatogram to give a solvent-corrected chromatogram for each solution. The areas of the main and impurity peaks were integrated. The peak areas were expressed as a percentage of the total constituent areas, such that the sum of all peak areas is equal to one (100%). Peaks were considered to be significant if their area percent was ≥0.1%.

#### 2.1.3. Reference Fluorophore Solutions

An aliquot of each fluorophore was dissolved in dimethyl sulfoxide (DMSO) to produce the corresponding reference solution. The concentration of each was determined gravimetrically. When exposed to air, all three dyes were found to absorb significant amounts of water, i.e., about 5% by weight. The dyes absorbed this water from the air over the course of minutes, making weighing problematic. Therefore, it was necessary to dry and then weigh the dyes under dry conditions using a glove bag under dry nitrogen in order to determine accurate mass concentrations for the dye solutions. The DMSO was purchased from Sigma-Aldrich, anhydrous, ≥99.9%, Cat. # 276855-2L, Lot # SHBK7268 (used with PO) and Lot # SHBK9388 (used with AF700 and AF750). The microbalance used for dye mass measurements was a Mettler Toledo XPR2U (S/N B735599409), which was internally calibrated. The balance used for solvent mass measurements was a Sauter RC4021 (S/N SV-03094). Both balances are also calibrated annually using external standard weights. All measurements were taken at a temperature of 22.0 ± 1.0 °C, 30% (outside glove bag) and 4% (inside glove bag) relative humidity and 1003 mbar atmospheric pressure. Three repetitions of each mass measurement were taken. All uncertainties given here are expanded with an expansion coefficient of *k* = 2.

### 2.2. Fluorescence Spectra

**Figure 1:**
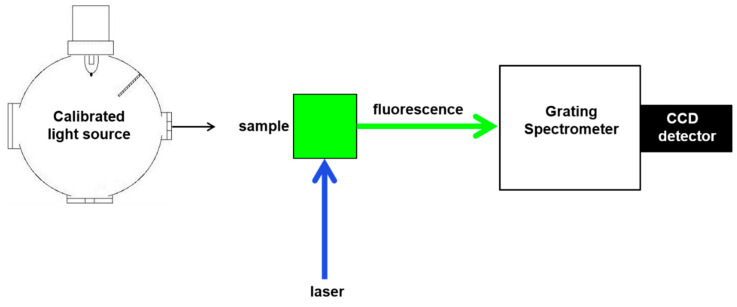
Schematic of the fluorescence spectrometer, with charge-coupled device (CCD) detector, used for the assignment of fluorescence intensities to calibration bead suspensions in equivalent reference fluorophore (ERF) units. The spectrometer was spectrally corrected for relative intensity of emission using a calibrated light source.

Each fluorescence spectrum was measured using a fluorescence spectrometer with a charge-coupled device (CCD) detector and laser excitation (see Figure 1). The relative radiometric accuracy as a function of wavelength of the signal (emission) detection system was corrected using a calibrated light source, traceable to the NIST realization of the International System of Units (SI) [10,11,12,13,14]. All fluorescence measurements were taken at 21 °C ± 1.0 °C using a 90° transmitting geometry with the excitation beam incident on and normal to one of the polished surfaces of the sample cuvette. All emission spectra were corrected for the responsivity of the detection system and normalized to the mean laser intensity measured over the same time period as each spectrum was taken. The excitation laser wavelengths available on the fluorescence spectrometer were 375, 405, 488, 561, 633 and 808 nm.

**Figure 2:**
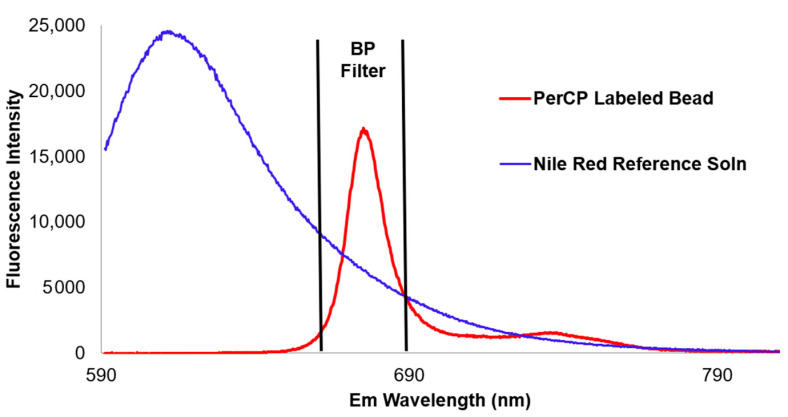
Fluorescence spectra of a reference fluorophore (Nile Red) and a calibration bead (PerCP labeled) and the bandpass (BP) filter spectral region over which the intensities were integrated for an ERF assignment.

Fluorescence intensity was measured by integrating a fluorescence spectrum (fluorescence intensity versus emission wavelength) over the fluorescence emission wavelength range determined by the emission filter corresponding to a particular fluorescence channel of a flow cytometer. Figure 2 shows an example where a Nile Red reference fluorophore solution was used to assign ERF units to the fluorescence intensity of the peridinin chlorophyll protein (PerCP) fluorescence channel of a flow cytometer using a PerCP-labeled bead suspension by integrating the fluorescence intensities of the two emission spectra over the range of the bandpass (BP) filter used for the PerCP channel. Note that calibration beads can be labeled with a single fluorescent dye designed to cover a single fluorescence channel, as shown here using PerCP, or many fluorescent dyes designed to cover most fluorescence channels, e.g., hard-dyed, multifluorophore beads.

### 2.3. Bead Number Concentration

#### 2.3.1. Micrometer-Sized Particles

A LO-based liquid particle counter was used to determine the bead number concentration of suspensions for beads with mean diameters greater than or equal to 2 μm [15,16]. The LO counter was a PAMAS model SVSS-C with an HCB-LD-25/25 sensor head, S/N U32757. Deionized, UV-sterilized and filtered water was used as a blank to measure the background of the instrument. A background of less than 20 mL^−1^ was achieved before samples were measured. The daily performance of the instrument was also verified by measuring the size and concentration of Thermo Count-Cal 5 μm beads. The measurement of a narrow distribution of sizes centered at 5 μm and a concentration within 10% of the manufacturer’s specification of 3000 mL^−1^ was recognized as a successful verification. The diameter of the Count-Cal beads is NIST traceable, but the concentration is not. Based on our estimates of accuracy and lot variation, a 10% uncertainty in the specified concentration was assumed. Each sample was shaken or vortexed for 10 s, then sonicated for 10 s, and then gently stirred by tipping the sealed container just before a set of ten measurements were collected.

A stock suspension of calibration beads was prepared at a nominal number concentration of 10^6^ mL^−1^ in the appropriate solvent for the bead. A sample suspension for LO measurements was prepared by diluting the stock solution with the appropriate solvent to a bead concentration of approximately 5000 mL^−1^.

Particle concentration was obtained by dividing a particle count by the sample volume. Traceability to the SI was assured by determining the confidence that all particles within the sample volume were counted, and by determining the actual sample volume. Qualification of the particle counter for high accuracy measurements and determination of uncertainties included (1) gravimetric calibration of volume, (2) pump volume dependence of particle counts to determine timing error, and (3) concentration dependence of particle counts to determine the linear range, correct for coincidence and determine sampling error due to bead adsorption to surfaces [15].

A flow cytometer was also used to confirm the LO-based bead concentration. This was done by using TruCount beads (BD Biosciences, San Jose, CA, USA) as an internal standard in the calibration bead suspension. A sample suspension for flow cytometer measurements was prepared by adding 100 μL of the stock solution to a TruCount tube and diluting with 400 μL of the appropriate solvent. Each TruCount tube contains a specified number of TruCount beads with a nominal value of 50,000 beads. The variation in the number of TruCount beads from tube to tube was estimated to be as much as 6%. The LO measurement was used to calculate the ERF values, because the uncertainties in the LO measurement were more thoroughly understood, such that the resulting bead concentration was traceable to the SI.

#### 2.3.2. Submicrometer Particles

Preliminary measurements of submicrometer beads with mean diameters ranging from 1 μm down to 80 nm were made in preparation for assigning ERF values to submicrometer calibration beads. The number concentrations of beads in suspensions were determined using a flow cytometer CytoFlex LX (Beckman Coulter, Brea, CA, USA) [17] and a microliter volume, next generation resistive pulse sensing (Next Gen RPS) instrument [18,19] (Spectradyne, Torrance, CA, USA), as well as other particle counting instruments designed for nanoparticle measurements, e.g., asymmetrical flow field-flow fractionation (AF4) [20] with dynamic light scattering (DLS) detection [21], nanoparticle tracking analysis (NTA) [22,23] and a standard resistive pulse sensing (RPS) instrument (Coulter Counter^®^, Beckman Coulter, Brea, CA, USA) [24]. NIST is also exploring a fluorescence-based virus counter [25] and a new way to use fluorescence microscopy for bead counting, the details of which have not yet been published.

## 3. Results

### 3.1. New Reference Fluorophores—Purity and Concentration Determinations

#### 3.1.1. qNMR Purity Determination

The absolute purity of PO was determined to be 0.9390 g/g (gram of PO with counter ion per gram of PO powder) and 0.7839(78) g/g (gram of PO without counter ion per gram of PO powder). Applying the latter purity value to the concentration gives a value of 20.10(23) mg PO/kg DMSO solution (3.724(43) × 10^−5^ mol/kg). Assuming a DMSO density of 1.0984(10) g/mL at 22 °C and using a molecular weight (MW) for PO (*w*/*o* counter ion) of 539.55 gives a value in moles per liter of 4.091(47) × 10^−5^ mol PO/L DMSO solution or about 41 μmol/L.

The absolute purity of AF700 was determined to be 0.9556 g/g (gram of AF700 with counter ion per gram of AF700 powder) and 0.7124(60) g/g (gram of AF700 without counter ion per gram of AF700 powder). Applying the latter purity value to the concentration gives a value of 20.15(17) mg AF700/kg DMSO solution (2.044(17) × 10^−5^ mol/kg). Assuming a DMSO density of 1.0984(10) g/mL at 22 °C and using a MW for AF700 (w/o counter ion) of 985.93 gives a value in moles per liter of 2.245(19) × 10^−5^ mol AF700/L DMSO solution or about 22 μmol/L.

The absolute purity of AF750 was determined to be 0.8762 g/g (gram of AF750 with counter ion per gram of AF750 powder) and 0.6054(81) g/g (gram of AF750 without counter ion per gram of AF750 powder). Applying the latter purity value to the concentration gives a value of 15.99(21) mg AF750/kg DMSO solution (1.812(24) × 10^−5^ mol/kg). Assuming a DMSO density of 1.0984(10) g/mL at 22 °C and using a MW for AF750 (w/o counter ion) of 882.02 gives a value in moles per liter of 1.991(27) × 10^−5^ mol AF750/L DMSO solution or about 20 μmol/L.

The purity by mass of each dye was determined with and without counter ion, because the stoichiometry found by qNMR for each dye was not an integer for the counter ion. The stoichiometric ratio for the counter ion versus the dye was determined to be 1.05, 3.3 and 3.9 for PO, AF700 and AF750, respectively. This implies that the molecular weight of the dye with counter ion could not be determined accurately; therefore, the molecular weight of the dye without counter ion was used to determine the purity in grams of dye per gram of solid sample. These values of 0.784 g/g ± 0.018 g/g, 0.712 g/g ± 0.012 g/g and 0.605 g/g ± 0.016 g/g were determined at a 95% confidence interval for PO, AF700 and AF750, respectively. Note that these are the purity and uncertainty reference values for the dyes.

#### 3.1.2. HPLC Purity Determination

For PO, two impurity peaks were observed with retention times after the main peak and a summed impurity area of 1.4%. For AF700, two impurity peaks were observed at retention times before the main peak and two more were observed after the main peak. The summed area of all four impurity peaks was 0.8%. For AF750, a single impurity peak was observed at a retention time before the main peak. The area of the impurity peak was 4.0%. The impurity peak was not observed when the detector monitored absorbance at 750 nm or fluorescence with 750 nm excitation. All HPLC purities are expressed here in mole percent and assume the extinction coefficients of all constituents are equal.

#### 3.1.3. Reference Fluorophore Solutions—Concentration Determination

The concentration and uncertainty at a 95% confidence level (U_95_), assuming *k* = 2, for each ampouled reference dye solution is given in Table 1. They are given as reference, not certified, values, according to NIST’s criteria, even though the magnitude of the uncertainties for purity and gravimetry are within acceptable limits for certified values, because the nature of the unknown impurities was not identified.

### 3.2. Fluorescence Intensity Measurements

**Figure 3:**
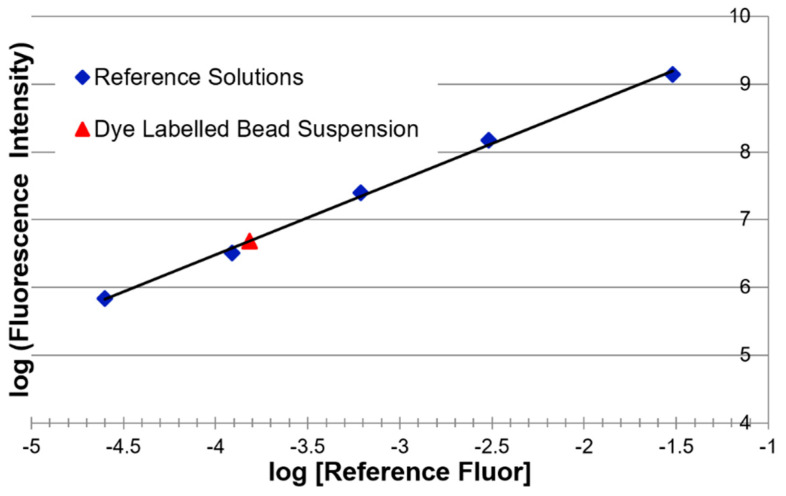
Calibration curve for fluorescence intensity as a function of reference fluorophore concentration. The curve is used to express the measured fluorescence intensity of the bead suspension in terms of the equivalent reference fluorophore concentration.

**Figure 4:**
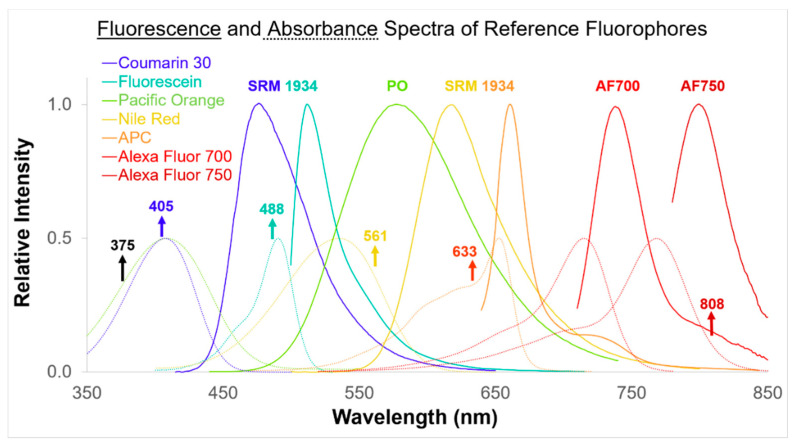
Fluorescence emission (solid lines) and absorbance (dotted lines) spectra of the reference fluorophores used to assign fluorescence intensities in ERF units. The upward arrows show the positions of the excitation lasers for the fluorescence spectrometer.

The integrated fluorescence intensity of the microparticles was measured in ERF units. This was achieved by first determining plots of integrated fluorescence intensity versus reference fluorophore concentration using serial dilutions of the appropriate reference solution, that is, one of the SRM 1934 constituents or a NIST internal RM fluorophore solution (see Figure 3). A straight line was fitted to the plot. This straight line defines the ERF intensity scale. The absorbance and emission spectra of all reference fluorophores and the excitation laser wavelengths available to the fluorescence spectrometer are shown in Figure 4. The absorbance spectrum determines which laser excitation (EX) wavelengths can be used to excite a reference fluorophore. The fluorescence emission (Em) spectrum defines the possible Em ranges that can be used with a reference fluorophore for ERF assignments.

**Figure 5:**
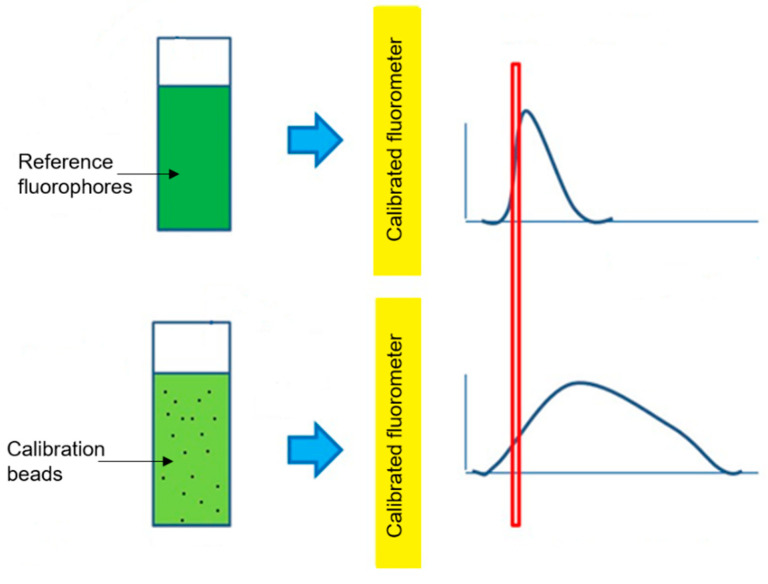
Schematic for measuring the fluorescence emission spectra of both reference fluorophore solutions and a calibration bead suspension using a calibrated fluorescence spectrometer (fluorometer) to determine the integrated fluorescence intensity of the bead suspension. The red rectangle shows the spectral range, defined by the emission bandpass filter of the fluorescence channel of a flow cytometer, over which the fluorescence spectra are integrated.

The integrated fluorescence intensity of a bead suspension was then measured using the same fluorescence spectrometer settings as those for the reference fluorophore (see Figure 5). The location of the suspension’s integrated fluorescence intensity on the fitted straight line was determined, giving the number of reference fluorophores needed to produce a fluorescence intensity equal to that of the bead suspension (see Figure 3). Note that the fluorescence intensities of both the bead suspensions being assigned and the corresponding reference fluorophore solutions must be linear with fluorophore concentration for intensity assignments to be useful. Bead manufacturers understand this and produce calibration beads at fluorophore concentrations in the linear range. NIST also ensures that reference fluorophore solutions are only used in their linear range for ERF assignments.

### 3.3. Bead Concentration Measurements

#### 3.3.1. Micrometer-sized Particle Counting

The number concentrations of calibration beads with mean diameters of 2, 3.5 and 10 µm were measured using LO, and the corresponding values measured using FCM with an internal standard were compared. The difference in the values between the two techniques was typically less than 5%. LO only differentiates particles based on size, whereas FCM differentiates based on fluorescence intensity. Since calibration beads are not completely homogeneous, there is typically a main fluorescence population with fluorescence intensities clustered very close together, representing 85% to 95% percent of the total bead population, and a smaller population with fluorescence intensities that are more scattered. The main population is gated during FCM data analysis and is the bead population of interest during calibration of a flow cytometer. Therefore, a flow cytometer is used to determine the mean fluorescence intensity (MFI) of the main and total populations and the corresponding MFI ratio.

#### 3.3.2. Submicrometer Particle Counting

The number concentrations and size distributions of beads with submicrometer diameters down to 80 nm were measured using several types of instruments and the results were compared between instruments. Although these results are preliminary, we expect these measurement techniques to enable ERF value assignments to be expanded to smaller diameter calibration beads. We will continue using all techniques listed in Section 2.3.2 to evaluate the comparability of results for submicrometer bead number concentrations.

### 3.4. ERF Assignments Per Bead

The mean ERF value for integrated fluorescence intensity of a single calibration bead was determined by dividing the ERF value for the bead suspension, as determined in Figure 3, by the number concentration of the bead suspension. This mean ERF value for the total bead population was multiplied by the MFI ratio of the main and total populations to determine the mean ERF value for the main population.

Each laser in a flow cytometer emits light at a single wavelength. The three most common laser wavelengths used in flow cytometers are at 405, 488 and 633 nm. Additional lasers at 375, 561 and 808 nm are also becoming more common in modern cytometers. The NIST fluorescence spectrometer used here is capable of assigning ERF values for fluorescence channels using all six of these EX wavelengths.

The Em range in flow cytometers is defined by the bandpass filter that the fluorescence passes through just before reaching the detector. Each fluorescence channel of a flow cytometer is defined by a single EX wavelength and a single Em range. The fluorescence channels that have been assigned by NIST are listed in Table 2, along with the reference fluorophore, EX wavelength (**λ_EX_**), center wavelength of the emission range (**λ_Em_**) and bandwidth of the fluorescence channel (**Δλ**) used for the assignment. Note that the EX wavelength and the Em range choices defining a fluorescence channel of a flow cytometer do not overlap, due to unwanted background that would be observed by the fluorescence detector at the EX wavelength from scattered laser light. NIST has not yet used the 808 nm laser to make an assignment even though the fluorometer used is capable of this. NIST has also not yet used the AF750 reference fluorophore for ERF assignments.

The total uncertainty in an ERF assignment was determined at the 95% confidence level (expansion coefficient *k* = 2) and ranged from 5% to 13% of the ERF value per bead.

## 4. Discussion

### 4.1. New Reference Fluorophores—Purity and Concentration Analyses

The qNMR dye purity value with counter ion was used to estimate the percent impurities for each new reference fluorophore. This gives impurity values of 6.1%, 4.4% and 13.4% by mass for PO, AF700 and AF750, respectively. HPLC suggested aromatic impurities in mole percent of 1.4%, 0.8% and 4.0%, respectively, which did not absorb or fluoresce light in the same spectral region as the main dye. The peaks from impurities observed in the NMR spectra agreed approximately with the HPLC values and did not account for the larger amounts of impurities determined by qNMR using an internal standard. These unknown impurities could be due to inorganics or solvent molecules bound to the dye that could not be driven off easily with heat. The identification of the unknown impurities will be pursued using ICP-MS and LC-MS in the future, contingent on more dye samples being obtained from the manufacturer. If the identity of the unknown impurities can be determined, then the reference values for dye purity may be upgraded to certified values in the future.

### 4.2. ERF Assignments Per Bead—Fluorescence Channels

Each calibration bead is designed to be excited at one or more laser wavelengths and to emit fluorescence at one or more Em ranges. The reference fluorophore used to assign a bead also needs to be chosen based on EX wavelength and Em range. The reference fluorophore needs to absorb significantly at the EX wavelength and emit a significant fluorescence intensity in the Em range. Consequently, an ERF value assigned applies only to a specific excitation-emission scheme for a fluorescence channel of a flow cytometer. The absorbance and fluorescence emission spectra for each reference fluorophore are given in Figure 4. This clarifies the fact that each reference fluorophore can be used to assign multiple fluorescence channels. NIST continues to develop additional reference fluorophores for fluorescence channels not yet covered, e.g., λ_EX_(nm)/λ_Em_(nm) = 405/421. Flow cytometer manufacturers have recently extended the emission range of their instruments further into the near infrared, due to decreased autofluorescence from microbes and other biological substances in this region, enabling improved quantitation of fluorescence intensities. The addition of 808 nm laser excitation and AF700 and AF750 reference fluorophores to the NIST fluorescence spectrometer reflects the emerging need for standardization in this spectral region.

### 4.3. Submicrometer Particle Counting

Based upon our preliminary results and reports in the literature, the size limit/range (a single number for size limit), typical sample volume, typical sample number concentration range and caveats related to submicrometer particle counting are given in Table 3 for different techniques presently being considered at NIST. LO is not an effective technique for submicrometer particles but is included for comparison since it can be a primary method for micrometer-sized particle number concentration (SI traceable) with sufficient instrument characterization. LO does require a significant dilution of the sample to prevent coincidence, which introduces error into the measurement.

All techniques listed in the table are single-particle detection methods, meaning that each particle is independently measured, except for AF4-DLS which measures scattered light to determine the motion of an ensemble of particles. Sizes measured by ensemble detection are typically only accurate for monodisperse samples, since multisize distributions often yield population-weighted, average values. The technique AF4 is used to separate particles by size in mixtures, allowing DLS detection to be more accurate. The technique DLS gives relative populations that are biased toward larger particles and is not quantitative for number concentration [26]. Using UV absorbance detection with AF4 can give quantitative particle number concentrations, which we intend to explore in the near future.

Electron microscopy (EM), including transmission electron microscopy (TEM) [27] and scanning electron microscopy (SEM) [28], are non-optical methods that can accurately measure the core size diameter and relative number concentration of particles in multisized particle mixtures. Their main drawback is the very difficult determination of the sample volume being counted. If the sample volume is not known with accuracy, then the number concentration will be inaccurate. Even for relative number concentration determinations, EM requires long sample collection times.

The technique NTA is an optical method (light scattering detection) that can accurately measure the hydrodynamic diameter of particles in multisized particle mixtures. It can also give an estimate of number concentration for monodisperse particle suspensions, based on a reference suspension (standard) of known number concentration used to calibrate the instrument, typically by the manufacturer. For multisized particle mixtures, the larger particles mask the effective detection of smaller particles, causing the smaller particles to be undercounted and their corresponding number concentrations to be inaccurate [26].

Resistive pulse sensing is an electrical impedance-based method that can accurately measure size of particles, even when polydispersed, but is limited to sizes greater than 200 nm and requires a significant dilution of the sample that introduces error, similar to LO. Next Gen RPS overcomes these deficiencies using microchip technology to decrease the size limit and sample volume while preserving accuracy. Similar to NTA, all RPS instruments require calibration using a reference suspension (standard) of known number concentration to give accurate number concentrations for samples.

Flow cytometry is an optical method (light scattering and fluorescence detection) that can yield detailed biochemical and cellular information, but needs an internal standard, such as a bead suspension with a known number concentration, to determine particle concentration. In order to be detected by scattering, particles also need to have a refractive index that is different from the flow fluid. Presently, general purpose flow cytometers that are commercially available have a size limit of about 80 nm [17]. Nano and quantum flow cytometers [25,29] are being developed to decrease this limit to 30 nm or smaller and typical sample volumes and number concentrations have not yet been established for these instruments.

**Table 3:** Particle Counting Techniques for Beads include light obscuration (LO), electron microscopy (EM), asymmetrical flow field-flow fractionation with dynamic light scattering (AF4-DLS), nanoparticle tracking analysis (NTA), resistive pulse sensing (RPS), next generation RPS (Next Gen RPS), flow cytometry (FCM), quantum FCM, and a virus counter.

Technique	Size Limit/Range (nm)	Sample Volume	Sample Concentration mL^−1^	Caveats	Ref.
LO	2000	15 mL	10^3^ to 10^4^	size limit dilution error	[15]
EM	1–100	10 mL	10^10^ to 10^12^	unknown volume collection time	[27,28]
AF4-DLS	2	10 mL	10 mg	not accurate	[20,26]
NTA	10–1000	12 mL	10^6^ to 10^9^	need standard	[22,23]
RPS	200	15 mL	10^4^ to 10^6^	need standard size limit dilution error	[24]
Next Gen RPS	60	10 mL	10^6^ to 10^10^	need standard	[18,19]
FCM	80	100 mL	10^5^ to 10^7^	need standard	[17]
Quantum FCM	30	N/D ^†^	N/D ^†^	N/D ^†^	[29]
Virus Counter	25–300	200 mL	10^5^ to 10^9^	virus specific	[25]

^†^ Not determined.

The virus counter is a specialized flow cytometer, designed to detect intact virus particles using a hydrodynamically focused nanostream and two fluorescence channels, one for nucleic acid detection and the other for capsid protein detection. No internal calibrant bead is required because the detected sample volume is determined by the instrument in real time. This allows virus concentrations to be determined in 5 to 6 min. The only caveat is that the instrument is designed for virus-specific detection, so its applications are limited. It has not yet been determined whether bead suspension concentrations can be measured using this type of instrument.

Fluorescence microscopy is also being developed at NIST as a technique for number concentration determination by clearly defining the sample volume in which the counted particles reside, in a similar way that a hemocytometer can be used, but with greater accuracy, precision and speed.

All of the single particle detection methods considered above give accurate particle counts for their specified size and number concentration range but can only yield accurate number concentrations for bead suspensions if the volume or mass of the sample is known. This can be done for EM with an elaborate sample delivery system. Most of the other techniques require an accurate, internal or reference standard for particle concentration, which also enables the determination of sample volume. The reference standards that are currently used for submicrometer particle concentration have been assigned values with unspecified uncertainties that are not traceable to the SI. This means that number concentration values assigned to samples using these “standards” may be inaccurate with uncertainties that cannot be determined. NIST is working on determining the sample volume of all of the above techniques with greater accuracy, enabling SI traceability of number concentration for submicrometer particles.

## 5. Conclusions

The highly accurate particle counting and fluorescence measurements that NIST uses to assign ERF-based fluorescence intensities of micrometer-sized calibration beads is improving the accuracy of calibration for FCM fluorescence channels with emission centered from 390 to 850 nm. NIST has assigned calibration beads, ranging in size from 2 to 10 µm, for more than 50 fluorescence channels using five different laser colors. The purity of reference fluorophores and the concentration of corresponding reference solutions used for ERF assignments has been determined with known uncertainties. NIST is in the process of expanding these assignments even further, with more laser colors and more reference fluorophores, intending to cover all fluorescence channels used in FCM.

In principle, there is no reason why the same strategy could not be used for ERF assignments of submicrometer beads, but techniques that can accurately measure number concentrations of these smaller beads need to be established first. NIST is collaborating with other stakeholders to establish these techniques. Submicrometer calibration beads with assigned ERF values would enable more accurate and standardized measurements to be made of nanoparticles and nanobioparticles, and establish measurement traceability in EV application fields. The Flow Cytometry Quantitation Consortium will continue to establish comparability, accuracy and measurement assurance in quantitative FCM assays.

## Figures and Tables

**Table 1:** Concentration and Uncertainty (**U_95_**) of NIST Reference Solutions for Pacific Orange (PO), Alexa Fluor 700 (**AF700**) and Alexa Fluor 750 (**AF750**), expressed using different units.

Units	PO	U_95_	AF700	U_95_	AF750	U_95_
mg/kg	20.10	0.46	20.15	0.34	15.99	0.43
mol/kg	3.724 × 10^−5^	8.6 × 10^−7^	2.044 × 10^−5^	3.4 × 10^−7^	1.812 × 10^−5^	4.9 × 10^−7^
mol/L	4.091 × 10^−5^	9.4 × 10^−7^	2.245 × 10^−5^	3.8 × 10^−7^	1.991 × 10^−5^	5.3 × 10^−7^

**Table 2:** NIST Assigned Fluorescence Channels with specified reference fluorophore (fluor), excitation wavelength (**λ_EX_**), center wavelength of the emission range (**λ_Em_**) and bandwidth of the fluorescence channel **Δλ**. Reference fluorophores include Coumarin 30 (C30), Pacific Orange (PO), fluorescein (FL), Nile Red (NR), allophycocyanin (APC) and Alexa Fluor 700 (AF700).

Reference Fluor (nm)	λ_EX_ (nm)/λ_Em_ (nm) (nm)	Δλ (nm) (nm)	Reference Fluor (nm)	λ_EX_ (nm)/λ_Em_ (nm)	Δλ (nm) (nm)
C30	375/450	45	NR	488/610	20
C30	375/525	40	NR	488/660	50
PO	375/675	30	NR	488/690	50
C30	405/440	50	NR	488/695	40
C30	405/450	45	NR	488/780	60
C30	405/450	50	NR	561/585	42
C30	405/512	25	NR	561/590	16
C30	405/525	40	NR	561/610	20
C30	405/525	50	NR	561/620	15
C30	405/530	30	NR	561/670	30
C30	405/605	40	NR	561/675	30
PO	405/610	20	NR	561/710	50
PO	405/615	24	NR	561/720	60
PO	405/660	10	NR	561/763	43
PO	405/670	30	NR	561/789	78
PO	405/763	43	APC	633/660	10
FL	488/525	20	APC	633/665	20
FL	488/525	35	APC	633/670	14
FL	488/525	40	APC	633/670	30
FL	488/525	50	APC	633/710	50
FL	488/530	30	AF 700	633/712	25
FL	488/530	40	AF 700	633/720	30
NR	488/574	26	AF 700	633/763	43
FL	488/585	40	APC	633/780	60
FL	488/585	42	AF 700	633/780	60
NR	488/593	52			

## NIST Disclaimer

Certain commercial equipment, instruments and materials are identified in this paper to specify an experimental procedure as completely as possible. In no case does the identification of particular equipment or materials manufacturer imply a recommendation or endorsement by the National Institute of Standards and Technology nor does it imply that the materials, instruments, or equipment are necessarily the best available for the purpose. This manuscript is a contribution of NIST, and therefore is not subject to copyright in the United States.
